# Plasminogen activator inhibitor-1 4G/5G polymorphism and retinopathy risk in type 2 diabetes: a meta-analysis

**DOI:** 10.1186/1741-7015-11-1

**Published:** 2013-01-02

**Authors:** Tengyue Zhang, Chong Pang, Ningdong Li, Elaine Zhou, Kanxing Zhao

**Affiliations:** 1 Tianjin Medical University, 22 Qixiangtai Road, Tianjin, 300020, PR China; 2 Tianjin Eye Hospital, Tianjin Key Laboratory of Ophthalmology and Visual Science, Tianjin Eye Institute, Clinical College of Ophthalmology Tianjin Medical University, 4 Gansu Road, Tianjin, 300020, PR China; 3 Department of Ophthalmology, Medical School of Yale University, 300 George Street, New Haven, 06511, USA; 4 Department of Thoracic Surgery, Tianjin Cancer Institute and Hospital, Huanhu West Road, Tianjin, 300060, PR China; 5 Saybrook College, Yale University, 242 Elm Street, New Haven, 06511, USA

**Keywords:** Diabetic retinopathy, meta-analysis, *PAI-1*, polymorphism, type 2 diabetes

## Abstract

**Background:**

Mounting evidence has suggested that plasminogen activator inhibitor-1 (PAI-1) is a candidate for increased risk of diabetic retinopathy. Studies have reported that insertion/deletion polymorphism in the *PAI-1 *gene may influence the risk of this disease. To comprehensively address this issue, we performed a meta-analysis to evaluate the association of *PAI-1 *4G/5G polymorphism with diabetic retinopathy in type 2 diabetes.

**Methods:**

Data were retrieved in a systematic manner and analyzed using Review Manager and STATA Statistical Software. Crude odds ratios (ORs) with 95% confidence intervals (CIs) were used to assess the strength of associations.

**Results:**

Nine studies with 1, 217 cases and 1, 459 controls were included. Allelic and genotypic comparisons between cases and controls were evaluated. Overall analysis suggests a marginal association of the 4G/5G polymorphism with diabetic retinopathy (for 4G versus 5G: OR 1.13, 95%CI 1.01 to 1.26; for 4G/4G versus 5G/5G: OR 1.30, 95%CI 1.04 to 1.64; for 4G/4G versus 5G/5G + 4G/5G: OR 1.26, 95%CI 1.05 to 1.52). In subgroup analysis by ethnicity, we found an association among the Caucasian population (for 4G versus 5G: OR 1.14, 95% CI 1.00 to 1.30; for 4G/4G versus 5G/5G: OR 1.33, 95%CI 1.02 to 1.74; for 4G/4G versus 5G/5G + 4G/5G: OR 1.41, 95%CI 1.13 to 1.77). When stratified by the average duration of diabetes, patients with diabetes histories longer than 10 years have an elevated susceptibility to diabetic retinopathy than those with shorter histories (for 4G/4G versus 5G/5G: OR 1.47, 95%CI 1.08 to 2.00). We also detected a higher risk in hospital-based studies (for 4G/4G versus 5G/5G+4G/5G: OR 1.27, 95%CI 1.02 to 1.57).

**Conclusions:**

The present meta-analysis suggested that 4G/5G polymorphism in the *PAI-1 *gene potentially increased the risk of diabetic retinopathy in type 2 diabetes and showed a discrepancy in different ethnicities. A higher susceptibility in patients with longer duration of diabetes (more than 10 years) indicated a gene-environment interaction in determining the risk of diabetic retinopathy.

## Background

Diabetic retinopathy (DR), the leading cause of blindness in the working population, is associated with a strong genetic predisposition, highlighted by the familial clustering of DR [1,2]. Several gene polymorphisms are associated with DR, such as in the methylenetetrahydrofolate reductase gene, endothelial nitric oxide synthase gene, manganese superoxide dismutase gene, vascular endothelial growth factor gene, receptor for advanced glycation end products gene, aldose reductase 2 gene and P-selectin gene, among others [3-9]. Plasminogen activator inhibitor-1 (PAI-1), the most important *in vivo *inhibitor of plasminogen activation, has also been implicated in DR. In addition to being involved in tissue repair and remodeling, PAI-1 plays a critical role in the regulation of intravascular fibrinolysis. In patients with type 2 diabetes (T2D), impaired fibrinolysis is involved in the pathogenesis of DR [10]. Increased PAI-1 expression has been associated with matrix accumulation [11] and the development of basement membrane thickening and pericyte loss, which are regarded as the earliest retinal pathohistological changes in DR in transgenic mice [10,12]. PAI-1 activity, which is affected by *PAI-1 *gene polymorphisms and metabolic determinants, is also elevated in DR [13]. Compared with other *PAI-1 *variants, the most significant variation in PAI-1 expression resides in a common single-base-pair guanine insertion/deletion polymorphism (4G/5G) within the promoter region of the *PAI-1 *gene at nucleotide position -675 [11,14]. Unlike the 5G allele that binds a transcription repressor protein, resulting in low PAI-1 expression, the 4G allele does not bind a transcription repressor, thus conferring a 'high PAI-1 expressor' nature to the allele [15].

Considering the potential influence on the individual risk for DR due to the insertion-deletion mutation of -675 4G/5G, many studies have explored the association between *PAI-1 *4G/5G and DR risk [16-24]. However, individual studies yielded inconsistent and even conflicting findings, which might be caused by the limitation of individual studies. To shed light on these contradictory results and to get a more precise evaluation of this association, we performed a meta-analysis of nine published case-control studies covering 1, 217 cases and 1, 459 controls.

## Methods

### Search strategy

In this meta-analysis, a comprehensive literature research of the US National Library of Medicine's PubMed database (to 1 May 2012) was conducted using research terms including 'plasminogen activator inhibitor-1', 'PAI-1', '4G/5G', 'polymorphism', 'type 2 diabetes', 'diabetic retinopathy' and the combined phrases to obtain all genetic studies on the relationship between *PAI-1 *4G/5G polymorphism and DR risk. There was no language limitation. We also hand-searched references of original studies or review articles on this topic to identify additional studies. The following criteria were used to select the eligible studies: they must be case-control studies on the association between *PAI-1 *4G/5G polymorphism and DR; and they must contain detailed and correct numbers of different genotypes for estimating an odds ratio (OR) with a 95% confidence interval (CI). When several publications reported on the same population data, the largest or most complete study was chosen. As a result, nine case-control studies were included in our meta-analysis.

### Data extraction

Two investigators independently assessed the articles for inclusion or exclusion, resolved disagreements, and attained consistency. For each eligible study, the following information was recorded: the first author's name, the year of publication, country of origin, ethnicity, total number of patients with DR and number of participants without DR (DWR) as well as the DR/DWR distribution in each *PAI-1 *genotype. Different ethnicities were categorized as Caucasian, Asian and Pima Indian. Sources of control were divided into population-based and hospital-based controls. The average duration of diabetes was separated into longer and shorter than 10 years.

### Statistical analysis

The strength of the relationship between *PAI-1 *4G/5G polymorphism and DR risk was assessed by calculating pooled ORs with 95% CIs. We evaluated the risk using the codominant model (4G/4G versus 5G/5G; 4G/5G versus 5G/5G), the dominant model (4G/4G + 4G/5G versus 5G/5G) and the recessive model (4G/4G versus 4G/5G + 5G/5G). Between-study heterogeneity was evaluated by χ^2^-based Q-test [25] and was considered significant if *P *< 0.10, in which case the random-effects model (the DerSimonian and Laird method [26]) was used to pool the data. If *P *> 0.10, the fixed-effects model (the Mantel-Haenszel method [27]) was selected. These two models provided similar results when between-studies heterogeneity was absent. Begg's funnel plot, a scatter plot of effect against a measure of study size, was generated as a visual aid for detecting bias or systematic heterogeneity [28]. Publication bias was assessed by the linear regression asymmetry test by Egger *et al*. (*P *< 0.05 considered representative of statistical significance [29]). Studies were categorized into subgroups based on ethnicity, average diabetes duration, and source of control. Hardy-Weinberg equilibrium was tested by the χ^2 ^test. All statistical analyses were performed in Review Manager (v.5.0; Oxford, England) and STATA Statistical Software (v.10.0; StataCorp, LP, College Station, TX). A two sided *P*-value < 0.05 was considered statistically significant.

## Results

### Eligible studies

**Figure 1:**
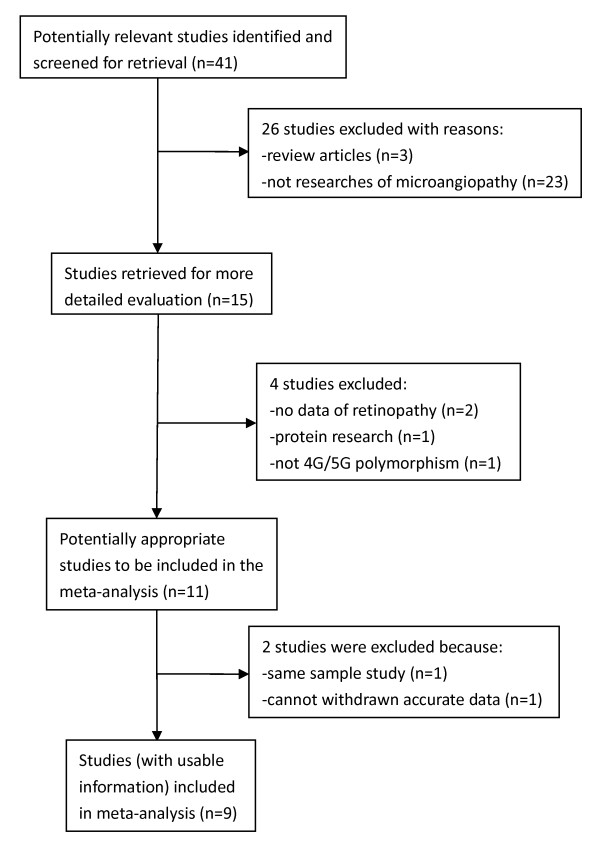
**Flow chart of included studies**.

In total, nine case-control studies including 1, 217 cases and 1, 459 controls were selected in our meta-analysis. A flow chart of the literature search, according to the Preferred Reporting Items for Systematic Reviews and Meta-Analyses guidelines [30], is shown in Figure 1. The main characteristics of these studies are shown in Table 1. Among these eligible publications, there were five studies of Caucasians, three studies of Asians and one study of Pima Indians. Four studies in which the average diabetes duration in all the subgroups was longer than 10 years were enrolled together and compared with the other five studies in which the diabetes duration was shorter than 10 years. There were two population-based studies and seven hospital-based studies. All studies used PCR methods for genotyping. The genotype distributions in the controls of all studies were in agreement with Hardy-Weinberg equilibrium. Thus, the sensitivity analysis was not performed in this study.

**Table 1:** Characteristics of case-control studies included in the meta-analysis.

First author	Year	Country	Ethnicity	Distribution of PAI genotypes						Sample Size (DR/DWR)	HWE	Reference Number
							
				4G/4G		4G/5G		5G/5G				
							
				DR	DWR	DR	DWR	DR	DWR			
Nagi	1997	U.S.A.	Pima Indian	14	18	44	45	12	39	70/102	0.43	[19]
Broch	1998	Spain	Caucasian	17	19	46	48	19	28	82/95	0.85	[21]
Wong	2000	China	East Asian	31	16	38	28	15	13	84/57	0.91	[23]
Santos	2003	Brazil	Caucasian	24	22	41	59	34	30	99/111	0.47	[16]
Globocnik-Petrovic	2003	Slovenia	Caucasian	39	25	58	40	27	15	124/80	0.89	[18]
Liu	2004	China	East Asian	15	27	26	49	15	15	56/91	0.36	[24]
Zietz	2004	Germany	Caucasian	48	112	55	173	28	73	131/358	0.68	[20]
Murata	2004	Japan	East Asian	78	43	86	35	24	14	188/92	0.14	[22]
Ezzidi	2009	Tunisia	Caucasian	77	54	167	242	139	177	383/473	0.56	[17]

DR: diabetic retinopathy; DWR: diabetes without retinopathy; HWE: Hardy-Weinberg equilibrium.

### Meta-analysis

**Figure 2:**
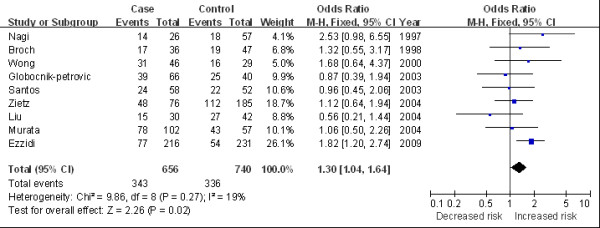
**Odds ratios of diabetic retinopathy associated with *PAI-1 *polymorphism (4G/4G versus 5G/5G) in overall studies**.

**Figure 3:**
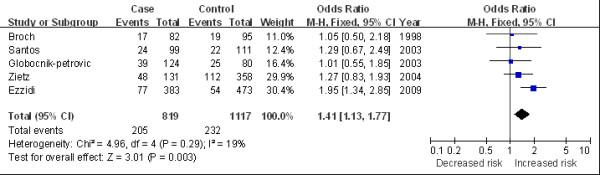
**Odds ratios of diabetic retinopathy associated with *PAI-1 *polymorphism (4G/4G versus 4G/5G + 5G/5G) in Caucasian populations**.

**Figure 4:**
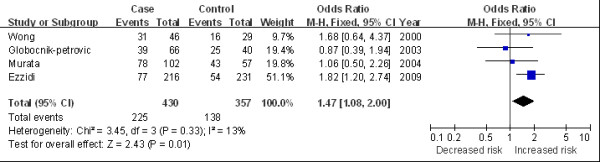
**Odds ratios of diabetic retinopathy associated with *PAI-1 *polymorphism (4G/4G versus 5G/5G) in average diabetes duration > 10 years**.

**Figure 5:**
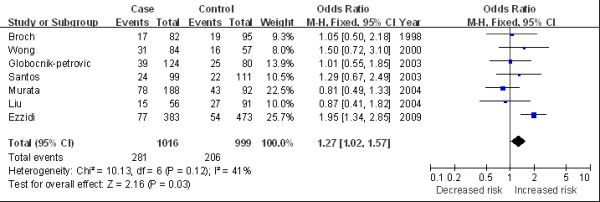
**Odds ratios of diabetic retinopathy associated with *PAI-1 *polymorphism (4G/4G versus 4G/5G + 5G/5G) in hospital-based population**.

The main results of this meta-analysis and the heterogeneity test are shown in Table 2. Overall, we found a marginally statistical significant association between 4G/4G and DR risk in overall population (for 4G/4G versus 5G/5G: OR 1.30, 95%CI 1.04 to 1.64, Figure 2; for 4G/4G versus 5G/5G + 4G/5G: OR 1.26, 95%CI 1.05 to 1.52). In the subgroup analysis by ethnicity, significantly increased risks were observed among the Caucasian population (for 4G/4G versus 5G/5G: OR 1.33, 95%CI 1.02 to 1.74; for 4G/4G versus 5G/5G + 4G/5G: OR 1.41, 95%CI 1.13 to 1.77, Figure 3). In the stratified analysis by average diabetes duration, the *PAI-1 *variation was found associated with elevated DR risk in patients with a duration of diabetes longer than 10 years (for 4G/4G versus 5G/5G: OR 1.47, 95%CI 1.08 to 2.00, Figure 4). We also detected an increasing risk in hospital-based studies (for 4G/4G versus 5G/5G + 4G/5G: OR 1.27, 95%CI 1.02 to 1.57, Figure 5).

**Table 2:** Summary of odds ratios and 95% confidence intervals of *PAI-1 *4G/5G polymorphism and diabetic retinopathy risk.

Comparisons	Number of studies	OR (95%CI)	*P^a^*	Alleles/genotypes (DR/DWR)
**4G/4G versus 5G/5G**				
**Total**	9	1.30 (1.04, 1.64)	0.27	1, 396 (656/740)
**Source of control**				
Population based	2	1.37 (0.85, 2.20)	0.15	344 (102/242)
Hospital based	7	1.28 (0.99, 1.67)	0.26	1, 052 (554/498)
**Ethnicity**				
Caucasian	5	1.33 (1.02, 1.74)	0.36	1, 007 (452/555)
Asian	3	1.00 (0.61, 1.65)	0.27	306 (178/128)
**Average diabetes duration**				
Longer than 10 years	4	1.47 (1.08, 2.00)	0.33	787 (430/357)
Shorter than 10 years	5	1.13 (0.80, 1.58)	0.27	609 (226/383)
**4G/5G versus 5G/5G**				
**Total**	9	1.01 (0.74, 1.37)	0.04	1, 970 (874/1, 123)
**Source of control**				
Population based	2	1.58 (0.42, 5.88)	0.00^**b**^	469 (139/330)
Hospital based	7	0.90 (0.72, 1.11)	0.39	1, 528 (735/793)
**Ethnicity**				
Caucasian	5	0.87 (0.70, 1.08)	0.55	1, 499 (614/885)
Asian	3	0.99 (0.61, 1.60)	0.22	358 (204/154)
**Average diabetes duration**				
Longer than 10 years	4	0.94 (0.73, 1.20)	0.62	1, 118 (554/564)
Shorter than 10 years	5	1.03 (0.57, 1.85)	0.00^**b**^	879 (320/559)
**4G/4G versus 5G/5G + 4G/5G**				
**Total**	9	1.26 (1.05, 1.52)	0.25	2, 676 (1, 217/1, 459)
**Source of control**				
Population based	2	1.25 (0.86, 1.80)	0.85	661 (201/460)
Hospital based	7	1.27 (1.02, 1.57)	0.12	2, 015 (1, 016/999)
**Ethnicity**				
Caucasian	5	1.41 (1.13, 1.77)	0.29	1, 936 (819/1117)
Asian	3	0.96 (0.67, 1.37)	0.37	568 (328/240)
**Average diabetes duration**				
Longer than 10 years	4	1.26 (0.80, 1.98)	0.03^**b**^	1, 481 (779/702)
Shorter than 10 years	5	1.16 (0.89, 1.53)	0.92	1, 195(438/757)
**4G/4G + 4G/5G versus 5G/5G**				
**Total**	9	1.07 (0.82, 1.39)	0.09^**b**^	2, 679 (1, 217/1, 459)
**Source of control**				
Population based	2	1.62 (0.52, 5.03)	0.01^**b**^	661 (201/460)
Hospital based	7	0.99 (0.81, 1.22)	0.46	2, 015 (1, 016/999)
**Ethnicity**				
Caucasian	5	0.99 (0.81, 1.21)	0.62	1, 936 (819/1, 117)
Asian	3	0.98 (0.62, 1.54)	0.22	568 (328/240)
**Average diabetes duration**				
Longer than 10 years	4	1.06 (0.84, 1.34)	0.81	1, 481 (779/702)
Shorter than 10 years	5	1.08 (0.64, 1.81)	0.01^**b**^	1, 195 (438/757)
**4G versus 5G**				
**Total**	9	1.13 (1.01, 1.26)	0.31	5, 364 (2, 446/2, 918)
**Source of control**				
Population based	2	1.23 (0.97, 1.56)	0.15	1, 322 (402/920)
Hospital based	7	1.10 (0.97, 1.25)	0.35	4, 042 (2, 044/1, 998)
**Ethnicity**				
Caucasian	5	1.14 (1.00, 1.30)	0.57	3, 884 (1, 650/2, 234)
Asian	3	0.97 (0.76, 1.25)	0.27	1, 136 (656/480)
**Average diabetes duration**				
Longer than 10 years	4	1.14 (0.98, 1.33)	0.42	2, 962 (15, 58/1, 404)
Shorter than 10 years	5	1.11 (0.94, 1.32)	0.16	2, 402 (888/1, 514)

**^a ^***P*-value for heterogeneity; **^b ^**Estimates for random-effects model. CI: confidence intervals; DR: diabetic retinopathy; DWR: diabetes without retinopathy; OR: odds ratio.

### Publication bias

**Figure 6:**
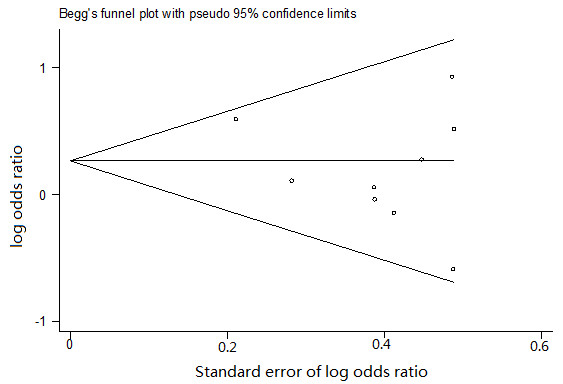
**Funnel plot of the meta-analysis of PAI-1 polymorphism and risk for diabetic retinopathy**.

The potential presence of publication bias was evaluated quantitatively by Begg's funnel plot and Egger's test. The Begg's funnel plot appeared symmetric. The Egger's test supported that there was no significant statistical evidence of publication bias for any of the four genetic models (for 4G/4G versus 5G/5G: *P *= 0.281; for 4G/4G versus 5G/5G + 4G/5G: *P *= 0.169; for 4G/5G versus 5G/5G: *P *= 0, 462; for 4G/4G + 4G/5G versus 5G/5G: *P *= 0.771). Figure 6 shows the shapes of the funnel plots of 4G/4G versus 5G/5G overall.

## Discussion

Unraveling the genes that contribute to the pathogenic risk of DR has been one of the major foci of basic research in DR over the past few decades. A large number of putative genes and genetic variants have been reported to be associated with higher risk of DR. A genome-wide association study performed on Mexican-Americans found several SNPs and genes associated with severe DR. None of these loci have been previously linked to DR or diabetes itself [31]. Another genome-wide association study on Taiwanese populations identified five loci not previously associated with DR susceptibility in T2D [32]. This suggests that, until now, no genes have achieved widespread acceptance as conferring high risk of DR in patients with T2D [33]. After a review of the meta-analyses of DR-related gene polymorphisms, only the C677T polymorphism in the methylenetetrahydrofolate reductase gene was detected to moderately augment the risk of DR in T2D overall [34,35]. The Gly82Ser polymorphism in the receptor for advanced glycation end products gene might be considered a risk factor for DR in Asian populations. Moderate evidence was founded for a correlation between the angiotensin-converting enzyme gene and proliferative DR [36]. However, neither the angiotensin-converting enzyme gene insertion/deletion [36-38] nor the vascular endothelial growth factor -634C/G gene [39] showed a significant relationship with DR, either overall or in ethnicity subgroups in meta-analysis so far.

The results of this meta-analysis suggest that *PAI-1 *4G/4G polymorphism was overall marginally significantly associated with DR risk in T2D. In the stratified analysis, significant associations were observed with Caucasian ethnicity, diabetes duration longer than 10 years and hospital-based studies. To the best of our knowledge, this is the first meta-analysis assessing the association between PAI-1 gene polymorphism and DR.

The earliest investigation into *PAI-1 *polymorphism and DR risk, reported by Nagi *et al*., revealed a positive relationship between the 4G allele of *PAI-1 *[19] and DR risk in Pima Indians, whose incidence of diabetes, particularly non-insulin-dependent diabetes, was extremely high [40,41]. However, in the subsequent studies in Caucasian populations, a trend of studies with a lack of association was suggested [16,18,20,21]. But in a recent larger case-control study in Tunisia that contained a total of 856 adult patients with T2D, Ezzidi *et al*. reported a significantly higher frequency of the 4G/4G genotype (OR 1.64, 95%CI 1.10 to 2.43), indicating 4G/4G in *PAI-1 *locus as a risk factor for DR [17]. All the studies in East Asian populations showed no relationship between 4G/5G polymorphism and DR risk [22-24]. Our meta-analysis confirmed that the 4G/4G genotype of the *PAI-1 *carried more risk in Caucasian but not Asian participants, even though the overall effect was positive. The differences in ethnic backgrounds, lifestyle, nutrition and living environment may partly explain this discrepancy [42]. We also found a marginally significant susceptibility to DR of T2D between *PAI-1 *4G/5G polymorphism to the population with longer duration of T2D. This subgroup analysis manifested a gene-environment interaction and highlighted the need for implementing rigorous case-selective criterion in future studies.

We also observed inconsistent results between hospital-based studies and population-based studies, which may be explained by the biases in hospital-based studies. Control cases in hospital-based studies may be less representative of the general population than those from population-based studies. Genes do not work in isolation; instead, complex molecular networks and cellular pathways are often involved in disease susceptibility [43]. Taking into account that DR is a complex disease with multifactorial, polygenic and environmental influences, a minor contributing pathogenic role of the *PAI-1 *polymorphism in specific cases in DR and in co-operation with other factors cannot be totally excluded.

Several potential limitations existed in our meta-analysis and our results should be interpreted with caution. First, as no correction for multiple testing was performed in this meta-analysis, false positive results may have been induced in some fraction because of the application of multiple statistical tests, which would increase the probability of type I errors. Second, our meta-analysis is based on unadjusted estimates because of a lack of original data. For example, the accurate disease time-course of individual patients was unavailable, which may potentially have affected the results where our classification criterion was according to the mean value of the diabetes duration. Third, this meta-analysis was limited by the small sample size - especially in subgroup analysis - though the Egger's test gave no publication bias [44]. Fourth, the existing studies lacked information about potential gene-gene interactions. Last, genotyping methods were different among selected studies, which might affect results. This discrepancy indicates the need to implement rigorous quality control procedures in future studies.

## Conclusions

Our meta-analysis suggests that *PAI-1 *polymorphism may be associated with elevated DR risk in patients with T2D, especially in the Caucasian population and in patients who have had diabetes for longer than 10 years. Future larger scale epidemiological investigation of this topic should be conducted to validate our findings.

## Abbreviations

CIs: confidence intervals; DR: diabetic retinopathy; DWR: diabetes without DR; ORs: odds ratios; PAI-1: plasminogen activator inhibitor-1; PCR: polymerase chain reaction; SNP: single nucleotide polymorphism; T2D: type 2 diabetes.

## Competing interests

The authors declare that they have no competing interests.

## Authors' contributions

KXZ contributed to the idea and design of this study and revised the manuscript. TYZ and CP carried out the screening procedure, performed the statistical analysis and drafted the manuscript. NDL participated in the design of the study, helped performed the statistical analysis and revised the manuscript. EZ helped to improve the English language and gave some suggestions to this manuscript. All authors read and approved the final manuscript.

## Pre-publication history

The pre-publication history for this paper can be accessed here:

http://www.biomedcentral.com/1741-7015/11/1/prepub
